# Cxcr1 mediates recruitment of neutrophils and supports proliferation of tumor-initiating astrocytes *in vivo*

**DOI:** 10.1038/s41598-018-31675-0

**Published:** 2018-09-05

**Authors:** Davalyn Powell, Meng Lou, Francisco Barros Becker, Anna Huttenlocher

**Affiliations:** 1 Department of Medical Microbiology and Immunology, University of Wisconsin-Madison, Madison, WI USA; 2 Department of Pediatrics, University of Wisconsin-Madison, Madison, WI USA

## Abstract

Neutrophils are first-responders to sites of infection and tissue damage including the inflamed tumor microenvironment. Increasing evidence suggests that crosstalk between tumors and neutrophils can affect the progression of established tumors. However, there is a gap in our understanding of the early events that lead to neutrophil recruitment to oncogene-transformed cells and how these pathways alter tumor progression. Here, we use optically transparent zebrafish larvae to probe the early signals that mediate neutrophil recruitment to Kras-transformed astrocytes. We show that zebrafish larvae with impaired neutrophil function exhibit reduced proliferation of transformed astrocytes supporting a critical role for tumor-associated neutrophils in the early progression of tumorigenesis. Moreover, using mutants and pharmacological inhibition, we show that the chemokine receptor Cxcr1 promotes neutrophil recruitment, proliferation of tumor-initiating cells, and neoplastic mass formation. These findings highlight the power of the larval zebrafish system to image and probe early events in the tumor-initiating microenvironment and demonstrate the potential for neutrophil recruitment signaling pathways such as Cxcl8-Cxcr1 as targets for anti-cancer therapies.

## Introduction

Neutrophils are critical first-responders to sites of infection and tissue damage and play an important role in host defense. Neutrophils also localize to the tumor microenvironment (TME) in a variety of human cancers and increased neutrophil infiltration and neutrophil-to-lymphocyte ratios in tumors often correlate with poor patient prognosis^1^. Therefore, there is increasing interest in understanding the role of neutrophils in the developing tumor microenvironment and how their presence alters adaptive immune responses. With recent advances in T-cell mediated immunotherapies, there is a need to better understand the role of innate leukocytes within the tumor and tumor-initiating niche, as well as the signals that mediate their recruitment to improve the efficacy of immunotherapy and patient outcomes.

Several recent studies have investigated the role of tumor-associated neutrophils (TANs) and how they influence the TME and cancer development (reviewed in^2–5^). For instance, there is evidence that neutrophils can modulate T cell tumoricidal activity by enhancing regulatory T cell recruitment and activity at the TME^6,7^. Neutrophils also secrete several factors including neutrophil elastase (NE), matrix metalloproteinases (MMPs), reactive oxygen species (ROS), vascular endothelial growth factor (VEGF), and others, which can modify the TME in favor of cancer cell growth and spread. On the other hand, an anti-tumor role for TANs has been reported in some models, and it is important to note that neutrophils exhibit phenotypic plasticity and heterogeneity which may contribute to a variety of effects in the TME^8–10^. Importantly, due to the constraints of available animal and cell culture models, few studies have investigated the role of neutrophils in early tumorigenesis or have identified signals that contribute to recruitment of neutrophils to tumor-initiating cells.

Neutrophils are highly migratory cells with the capacity to move within the blood stream and migrate interstitially to rapidly localize to sites of infection or injury. Several signaling pathways are involved in neutrophil chemotaxis and recruitment, many of which are also upregulated in the TME. One of the primary chemotactic pathways that regulates neutrophil migration is the CXCL8 (IL8)-CXCR1/2 pathway. In humans, CXCL8 is a chemokine which can be produced by many different cell types in response to various cytokines, ROS, pathogens, and other environmental stresses^11^. Importantly, CXCL8 is conserved between human and zebrafish, but has not been identified in mice^12^. The primary receptors for CXCL8 are CXCR1 and CXCR2 which are expressed on the surface of neutrophils and regulate chemotaxis. CXCL8 is upregulated in many cancers including solid tumors (brain, breast, colon, gastric, lung, and others) and blood cancers (AML, CLL, Hodgkin’s lymphoma) and high levels of CXCL8 expression are often linked with disease progression^13^. CXCL8 can function in both a paracrine manner to alter the immune composition of the TME as well as in an autocrine manner to regulate tumor cell EMT and invasion^14^. The role of CXCL8 and its homologs in recruiting neutrophils to established tumors has been demonstrated in various Ras-driven cancer models^15,16^, however its role in recruiting neutrophils to tumor-initiating cells and the role of specific receptors is unclear.

To address the role of neutrophils and chemotactic signaling in the pre-tumor niche, we used an emerging cancer model system, the zebrafish larvae, to model glioblastoma initiation and progression. Glioblastoma is an aggressive cancer type originating from astrocytes and is marked by high levels of neutrophilic inflammation^17^ with high neutrophil-to-lymphocyte ratios and neutrophil activity correlating with enhanced tumor progression and poor patient outcomes^18–20^, however little is known about neutrophil interaction with early transformed astrocytes *in vivo*. The zebrafish larval model allows for unparalleled non-invasive imaging of the early stages of tumor initiation as well as interactions between tumor-initiating cells and immune cells. Starting at 2 days post-fertilization (dpf), zebrafish larvae have a functional innate immune system comprising macrophages and neutrophils, however the adaptive immune system including B and T cells does not fully develop until approximately 2 weeks of development^21–23^. This early developmental window allows us to investigate the specific contribution of innate immune cells to the tumor-initiating niche. Using a transgenic model of glioblastoma initiation, we observed that neutrophils are highly recruited into the zebrafish hindbrain following expression of oncogenic Kras in astrocytes and that blocking neutrophil motility and function reduces the proliferative capabilities of Kras-expressing astrocytes. Additionally, we determined that the chemokine receptor Cxcr1 contributes to neutrophil recruitment into the tumor-initiating site while, similar to the wound microenvironment^24^, Cxcr2 is dispensable for neutrophil recruitment. Pharmacological inhibition of Cxcr1/2 reduces proliferation and mass formation within the brain. Altogether, these data suggest that the pro-neutrophil recruitment signaling axes, including Cxcl8-Cxcr1, are potential therapeutic targets of interest for glioblastoma and other inflammatory cancers.

## Materials and Methods

### Zebrafish care and maintenance

All adult and larval zebrafish were maintained according to protocols approved by the University of Wisconsin-Madison IACUC and in accordance with current guidelines and recommendations (ZFIN.org). Zebrafish embryos were treated with 0.003% phenylthiourea (PTU) in E3 media beginning at 24 hpf to prevent pigment formation. Zebrafish lines available via Zebrafish International Resource Center (ZIRC) include AB (wildtype), Tg(*mpx*:*mCherry*)^uwm7^, Tg(*mpx*:*mCherry*,*rac2*^D57N^)^zf307^, and *tp53*^zdf1^. Progeny from *cxcr1* heterozygote in-cross were genotyped using the following primers and imaged on 3% MetaPhor agarose gel (7 bp deletion): cxcr1F- 5′ CCTGGTAGACTTCCACGAGTTC 3′, cxcr1R- 5′ TGAAATGTTGACAGCGGATG 3′^24^. As no significant difference was observed between WT and heterozygous siblings, these groups were combined for analysis of neutrophil recruitment and proliferation.

### Plasmid expression

GFP-tagged human Kras^G12V^ or GFP alone was cloned under the *gfap* promoter (^25^ both gifts from Michael Taylor, UW-Madison) via Gateway (ThermoFisher) cloning into the pDest-tol2 backbone. To generate WT controls, G12 point mutation was introduced via site-directed mutagenesis (QuikChange Lightning, Agilent) via the following primers: F 5′ CACTCTTGCCTACGCCACCAGCTCCAACTACCAC 3′ and R 5′ GTGGTAGTTGGAGCTGGTGGCGTAGGCAAGAGTG 3′. mCherry-tagged Histone H2B^26^ was cloned under the *gfap* promoter using the following primers: F 5′ AAGGCTAGCAAGATCTGCTCGAGGGTACCCTCGAGGTAAGGA 3′ and R 5′ CTTGCTCACCATGGTGGCGGGTACCGGTGGCGACCGGGCTGC 3′. 25–38 ng of plasmid DNA + 25 ng of *transposase* mRNA was injected into the single-cell stage embryo. Embryos were screened for fluorescent protein expression at 72 hours post-fertilization.

### Live imaging of zebrafish larvae

For live imaging assays, zebrafish larvae were anesthetized at 3 dpf with 0.2 mg/mL Tricaine (MS222/ethyl 3-aminobenzoate; Sigma-Aldrich) in E3 media. For time-lapse imaging, larvae were mounted in 1% low-melting temperature agarose in E3 + Tricaine. Images were acquired by spinning disk confocal microscope (Yokogawa CSU-X) with a confocal scanhead on a Zeiss Observer Z.1 inverted microscope with NA 0.5/20X objective. Z-stack acquisition was restricted to the hindbrain in the Z- (lateral) plane. Images were processed using ZEN software (ZEN 2.3 Blue) and analyzed using Fiji (imagej.net/Fiji). Maximum intensity projections are shown unless otherwise labeled.

### Characterization of morphology and quantification of neutrophil recruitment and proliferation

To characterize transformed cell morphology, live imaging of Kras-expressing larval hindbrains was captured as above. ZEN software was used to analyze both maximum intensity projections and stacks for 3D morphology of GFP-positive cells. Larvae were categorized based on the most severe cell morphology observed in the hindbrain region (i.e. the presence of a single mass was categorized as “presence of masses”). To quantify neutrophil recruitment, live images of the hindbrain and surrounding tissue were acquired as above. *mpx*:mCherry-positive neutrophils within the field of view were counted and then normalized to the GFP-positive area. GFP area was measured in Fiji using the Image > Adjust > Threshold function to manually remove background autofluorescence and the Analyze > Measure function. To quantify proliferation of tumor-initiating astrocytes, larvae were fixed and antibody stained as described below and then imaged via confocal microscopy similar to live-imaging experiments. In each acquired stack, cells which were both GFP and pH3 positive in optical sections were counted and then normalized to total GFP-positive area as described above.

### Quantitative PCR and antibody staining

For qRT-PCR assays, 15–20 whole larvae per condition were pooled and placed in RNAlater (Ambion) at 4 °C for up to 2 weeks prior to mRNA isolation by Qiagen RNeasy Micro Kit and cDNA synthesis by SuperScript™ III First-Strand Synthesis System (ThermoFisher). qPCR was performed on a LightCycler 96 (Roche) using the FastStart Essential DNA Green Master kit (Roche) and previously published primers: *rps11* (housekeeping gene), *cxcl8a*, *cxcl8b*, *il1b*^27^; *vimentin*, *mmp9*^28^; and *cdh1*, *cdh2*^29^. For whole mount antibody staining, larvae at 3 dpf were fixed with 1.5% formaldehyde in 0.1 M PIPES, 1.0 mM MgSO4, and 2 mM EGTA overnight at 4 °C as previously described^30^ and immunolabeled with 1:300 rabbit anti-phospho-Histone H3 antibody (Millipore 06–570) or rabbit anti-Cdh2 antibody (GeneTex GTX125885) in 1% BSA overnight at 4 °C followed by anti-rabbit secondary antibodies at 1:250 (DyLight^TM^) in 1% BSA overnight at 4 °C. Stained larvae were imaged as above.

### Morpholino-mediated knockdown

3 nL of morpholino (Gene Tools) was injected into one-cell-stage embryos. Morpholino sequences and concentrations are as follows: standard control morpholino (5′-CCTCTTACCTCAGTTACAATTTATA-3′, 100 µM), *cxcr1* morpholino targeting the e1-i1 boundary (5′-TGTCAGGATACTAAACTTACCAGTC-3′, 75 µM)^31^.

### Drug treatments

Dechorionated larvae were treated with 1 μM SB225002 (Calbiochem; EMD Millipore) at 2.5 dpf in E3 with 0.02% DMSO for 16 hours prior to imaging. For drug treatments lasting until 6 dpf, media was exchanged with fresh E3 + SB225002 every 24 hours. Vehicle control samples were treated with 0.02% DMSO with daily media exchange.

### Graphical representation and statistical analysis

All data comprise at least three independent experimental replicates unless otherwise noted, with n reported as total number of individual larvae analyzed across all replicates. Experimental conditions are plotted in terms of mean and 95% confidence interval. In scatter plots, each dot refers to a single larva with all larvae (total n) reported across all replicates. Unpaired *t*-test was performed unless otherwise indicated; Chi-square analysis was performed for contingency analysis with a Bonferroni correction for multiple comparisons. Statistical analyses and graphical representations were made in GraphPad Prism version 6.

## Results

### Zebrafish model of glioblastoma initiation

**Figure 1:**
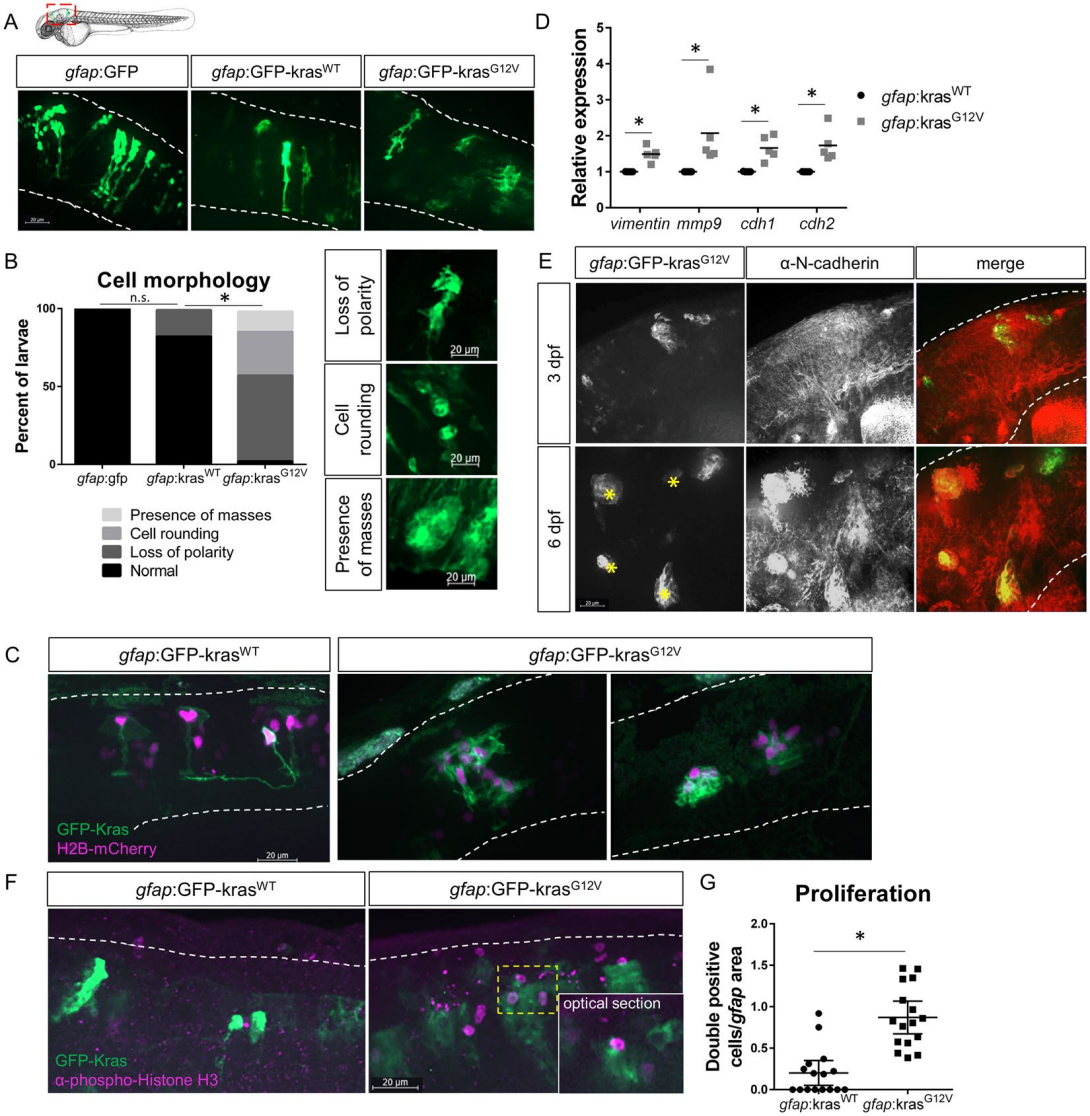
Expression of oncogenic Kras from an astrocyte-specific promoter drives transformation in the larval hindbrain. Live-imaging of mosaic *gfap*:GFP, *gfap*:GFP-kras^WT^, and *gfap*:GFP-kras^G12V^ expression at 3 days post-fertilization (dpf) in the larval zebrafish hindbrain (**A**). Red dashed outline in schematic of A shows approximate field of view and image orientation for all figures. White dashed lines in representative images denote the hindbrain region of interest in all figures. Morphology of expressing cells was categorized based on characteristics observed throughout the population of injected larvae and larvae were assigned to category of most severe cell morphology identified (**B**, n = 19 *gfap*:GFP, 17 *gfap*:kras^WT^, and 38 *gfap*:kras^G12V^ larvae; Chi-squared analysis performed). *gfap*:H2B-mCherry was mosaically co-expressed with *gfap*:kras plasmids to label nuclei of Kras-expressing cells (**C**). qRT-PCR was performed on whole-larvae isolates for EMT genes including *vimentin*, *mmp9*, *cdh1* (*e-cadherin*), and *cdh2* (*n-cadherin*). Cq were normalized to housekeeping gene *rps11* and then normalized to kras^WT^ condition (**D**, n = 5 kras^WT^ and 5 kras^G12V^ replicates). Larvae were fixed at 3 dpf or 6 dpf and immunostained for N-cadherin (**E**). N-cadherin co-localization was observed in *gfap*:kras^G12V^-expressing masses at 6 dpf (yellow asterisks). Kras-expressing larvae were fixed at 3 dpf and immunostained for proliferation marker phospho-Histone H3 (pH3, **F**). pH3/GFP double positive cells were quantified using confocal optical sectioning (single optical slice from yellow dashed box shown in inset) and normalized to the total *gfap*-expressing area (G, n = 16 larvae/condition). *p < 0.05.

To investigate the role of neutrophils in tumor-initiation, we followed up on a published zebrafish model of glioblastoma that used the Gal4-UAS system to drive oncogenic human Kras^G12V^ under the zebrafish astrocyte-specific *gfap* promoter and formed tumors as early as 6 months post-fertilization^25^. To image early tumor-initiation of a subset of astrocytes, we injected plasmids expressing GFP-tagged human Kras^G12V^ or wildtype Kras (Kras^WT^) under the control of the *gfap* promoter into single-cell embryos, driving robust mosaic expression in the zebrafish brain by 2 days post-fertilization (dpf). For this study, we focused on 3 dpf, a time point by which neutrophils are mature and migrating interstitially throughout the head region adjacent to the hindbrain (Fig. 1A). Live imaging of larvae demonstrates that with expression of oncogenic Kras^G12V^, the morphology of astrocytes is altered compared to cells expressing GFP alone or cells expressing wildtype Kras (Fig. 1A,B). Normal astrocytes within the zebrafish hindbrain exhibit a distinctive dorsal-ventral polarity and stellate morphology which is largely maintained in Kras^WT^-expressing cells. Kras^G12V^-expressing cells exhibit a loss of polarity, cell rounding, and occasionally the formation of large, multi-cellular masses (in ~13% of larvae). Co-expression of a construct encoding *gfap*-driven H2B-mCherry to label astrocyte nuclei further demonstrates the altered morphology and multi-cellular mass formation of Kras^G12V^-expressing cells (Fig. 1C). These changes in cell shape are accompanied by changes in gene expression. qRT-PCR of whole larvae revealed upregulation of EMT and adhesion genes including *vimentin*, *mmp9*, *cdh1* (*E-cadherin*), and *cdh2* (*N-cadherin*) at 3 dpf in *gfap*:Kras^G12V^-expressing larvae (Fig. 1D). In larvae which formed large masses by 6 dpf, N-cadherin-positive masses were also identified in a subset of larvae (<10%), consistent with EMT-like changes reported for glioblastoma^32,33^ (Fig. 1E). To further demonstrate oncogenic Kras-driven transformation of larval astrocytes, we assessed proliferation of Kras^G12V^-expressing cells. We performed whole mount immunofluorescent staining for proliferation marker phospho-histone H3 (pH3)^34^ in *gfap*:Kras^G12V^-expressing larval hindbrains at 3 dpf and imaged using confocal microscopy (Fig. 1F). Quantification of Kras^G12V^/pH3 double-positive cells was performed on optical sections revealing significantly increased proliferation of cells expressing oncogenic Kras (Fig. 1G). Counts were normalized to GFP-expression area in all experiments to account for the mosaic transformation pattern. At this developmental stage, non-GFP-Kras^G12V^ positive proliferating cells (pH3-positive only) are also observed within the hindbrain, however no significant differences in this population of cells was observed between experimental groups (data not shown). These data suggest that as early as 3 dpf, Kras^G12V^ drives oncogenic changes in zebrafish astrocytes which model early tumor-initiating events.

### Neutrophils are recruited to the tumor-initiation niche

**Figure 2:**
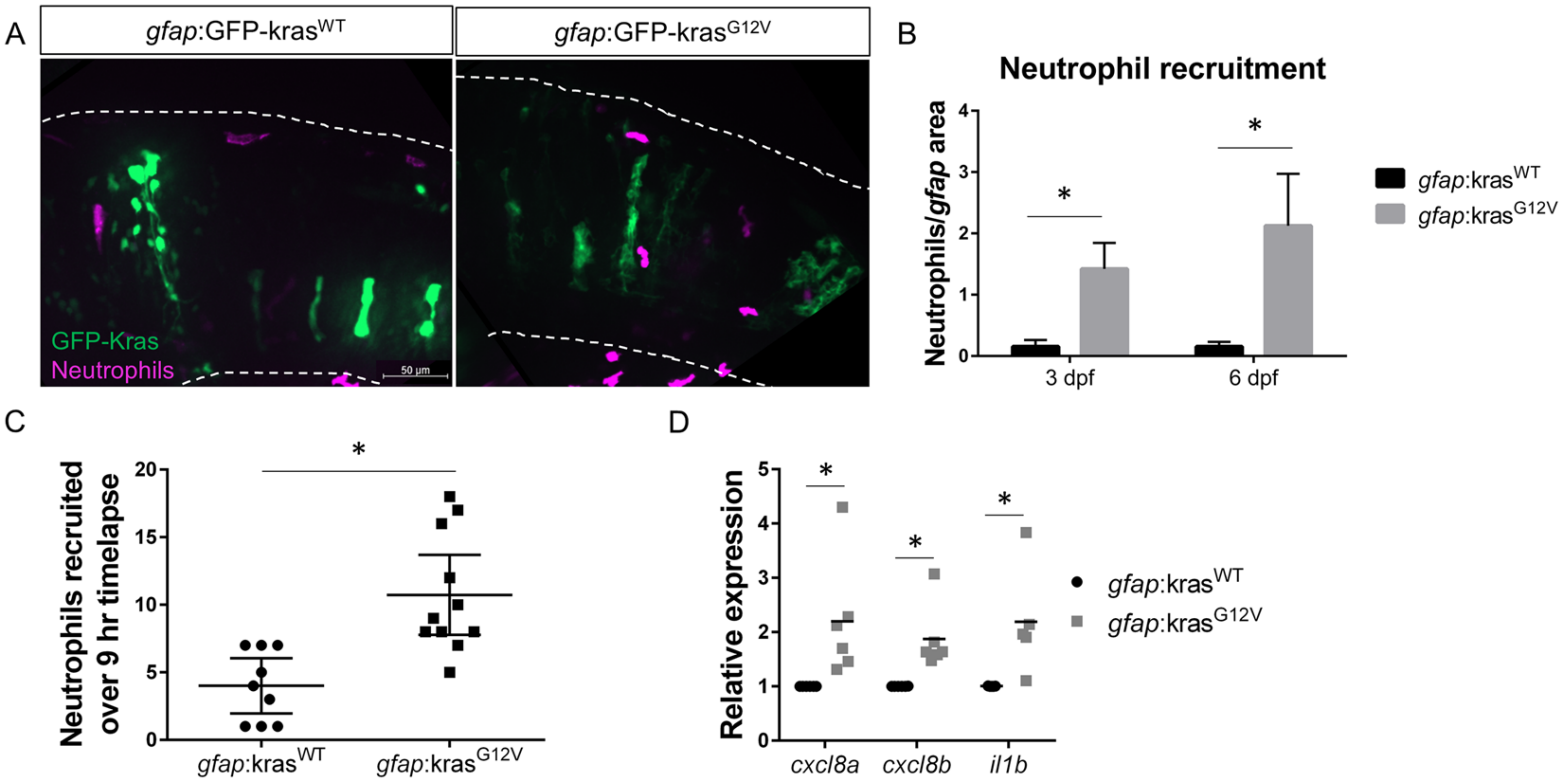
Neutrophils are recruited to the tumor-initiating microenvironment. Live-imaging of neutrophils in the hindbrain of Tg(*mpx*:mCherry) larvae expressing *gfap*:kras^WT^ or kras^G12V^ at 3 dpf (**A**). Neutrophils within the field of view were quantified and normalized to the total *gfap*-expressing area within the same field of view at 3 and 6 dpf (**B**, n = 24 kras^WT^ 3dpf, 42 kras^G12V^ 3 dpf, 24 kras^WT^ 6 dpf, and 30 kras^G12V^ 6 dpf larvae). Live time-lapse imaging was performed for 9 hours beginning at 3–3.5 dpf and total neutrophils recruited to the hindbrain during the period of imaging were quantified (**C**, n = 9 kras^WT^ and 11 kras^G12V^ larvae). qRT-PCR was performed on whole-larvae mRNA isolates for several neutrophil recruiting chemokines implicated in the tumor microenvironment including *cxcl8a*, *cxcl8b*, and *il1b* (**D**, n = 6 replicates for *cxcl8a/b*, 5 replicates for *il1b*, normalized as in Fig. 1D). *p < 0.05.

While neutrophils are frequently found in established tumors, their presence at sites of tumor initiation is less well characterized. In a zebrafish model of Hras-driven epithelial cell transformation, it was observed that neutrophils were recruited to transformed cells within 48 hours of Hras expression^28^. The zebrafish hindbrain, which is the site of astrocyte transformation in our model, is an excellent niche to study neutrophil recruitment since neutrophils are rarely found in uninfected or undamaged brain tissue. When Kras^WT^ is expressed in astrocytes, neutrophil recruitment to the hindbrain, as observed by live imaging of neutrophil-specific *mpx*:mCherry transgenic larvae, is minimal (Fig. 2A,B). However, when Kras^G12V^ is expressed, neutrophils are recruited to the brain and surrounding microenvironment at 3 dpf. Live, time-lapse imaging of the hindbrain demonstrates dynamic trafficking of neutrophils into the tumor-initiating and surrounding microenvironments, resulting in prolonged neutrophil recruitment in *gfap*:Kras^G12V^-expressing larvae (Fig. 2C, Supplemental Movies 1–2). Interestingly, neutrophils are rarely observed to pause within the tumor-initiating microenvironment and do not appear to directly contact transformed cells for any notable period of time. To determine which pathways might be involved in neutrophil recruitment to the tumor-initiating microenvironment, we performed qRT-PCR for several inflammatory cytokines. We determined that *gfap*:Kras^G12V^ results in an upregulation of the pro-inflammatory cytokine *il1b* (IL-1β) as well as the neutrophil chemokines *cxcl8a* (IL-8a) and *cxcl8b* (IL-8b) at 3 dpf (Fig. 2D). These results suggest that the tumor-initiating microenvironment produces inflammatory signals that recruit neutrophils at very early stages during transformation.

### Neutrophil recruitment to the tumor-initiating microenvironment enhances proliferation of transformed cells

**Figure 3:**
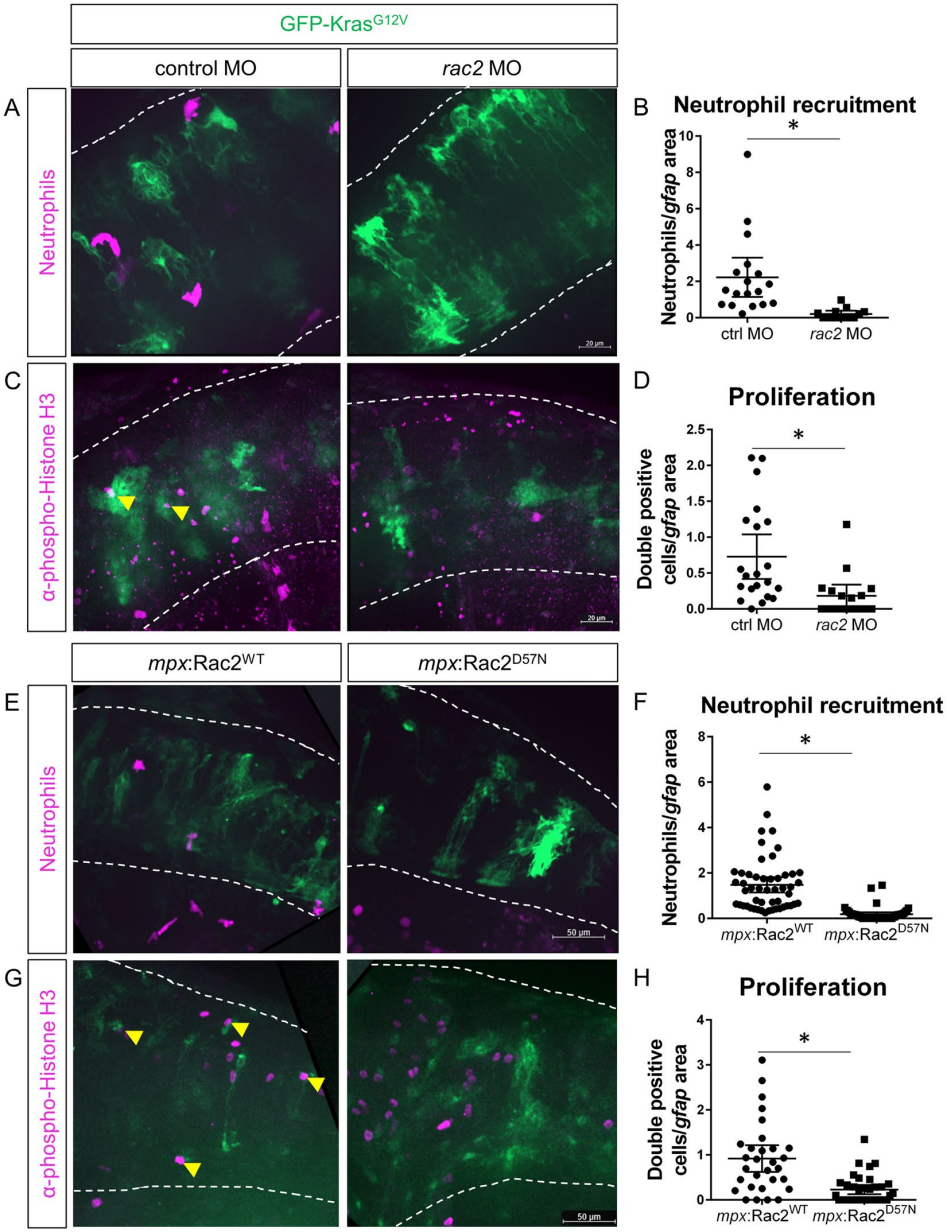
Blocking neutrophil motility reduces proliferation of tumor-initiating cells. Wildtype or Tg(*mpx*:mCherry) embryos were co-injected with *gfap*:GFP-kras^G12V^ plasmid and *rac2* or standard control morpholino (MO). Larvae were live-imaged for neutrophil recruitment at 3 dpf (**A**) and quantified for neutrophils within the field of view normalized to GFP-expression area (**B**, n = 13 control MO and 18 *rac2* MO larvae). Larvae were also fixed at 3 dpf and immunostained for phospho-Histone H3 (**C**). Double-positive (pH3 and GFP) cells were quantified by optical sectioning and normalized as above (**D**, yellow arrowheads in **C**, n = 21 control MO and 17 *rac2* MO larvae). Tg(*mpx*:mCherry-2a-Rac2^WT^) or Tg(*mpx*:mCherry-2a-Rac2^D57N^) embryos were injected with *gfap*:GFP-kras^G12V^ and live-imaged at 3 dpf (**E**). mCherry-positive neutrophils were quantified and normalized as above (**F**, n = 52 Rac2^WT^ and 46 Rac2^D57N^ larvae). Larvae were fixed at 3 dpf and immunostained for phospho-Histone H3 (**G**). pH3-GFP double positive cells were quantified by optical section and normalized as above (H, n = 30 Rac2^WT^ and 37 Rac2^D57N^ larvae). *p < 0.05.

To determine the effect of neutrophil recruitment on tumor-initiation, we employed previously characterized zebrafish models of impaired neutrophil migration and function and characterized early proliferation of transformed astrocytes. We first used whole larvae morpholino-mediated knockdown of the small Rho-GTPase, Rac2, which is required for neutrophil motility and recruitment to sites of tissue injury and infection in zebrafish larvae^35^. We co-injected the *rac2* morpholino (MO) with *gfap*:GFP-Kras^G12V^ into Tg(*mpx*:mCherry) larvae and assessed neutrophil recruitment to the hindbrain. As expected, at 3 dpf, neutrophil recruitment to the hindbrain was almost completely ablated in *rac2* knockdown larvae, demonstrating efficacy of this model in preventing neutrophil migration to the site of tumor-initiation (Fig. 3A,B). We next assayed for transformed cell proliferation following *rac2* knockdown. Interestingly, in the *rac2* knockdown, Kras^G12V^-expressing astrocytes exhibited significantly reduced proliferation compared to controls (Fig. 3C,D). To determine if this result was due to *rac2* regulating neutrophil motility or through transformed cell-intrinsic effects, we used neutrophil-specific expression of a dominant-negative Rac2 mutation, *mpx*:Rac2^D57N^, which similarly blocks neutrophil recruitment into tissues^35^. As seen in the whole-larvae knockdown, neutrophils were unable to migrate into the hindbrain of *gfap*:Kras^G12V^-expressing larvae (Fig. 3E,F). pH3 staining in the *mpx*:Rac2^D57N^ larvae also revealed that the transformed cells exhibited reduced proliferation in the absence of neutrophil infiltration into the microenvironment (Fig. 3G,H). These data demonstrate that neutrophil recruitment and presence in the tumor-initiating microenvironment enhances the proliferation of tumor-initiating cells within the brain.

### Chemokine signaling through Cxcr1 promotes neutrophil recruitment to the tumor-initiating microenvironment

**Figure 4:**
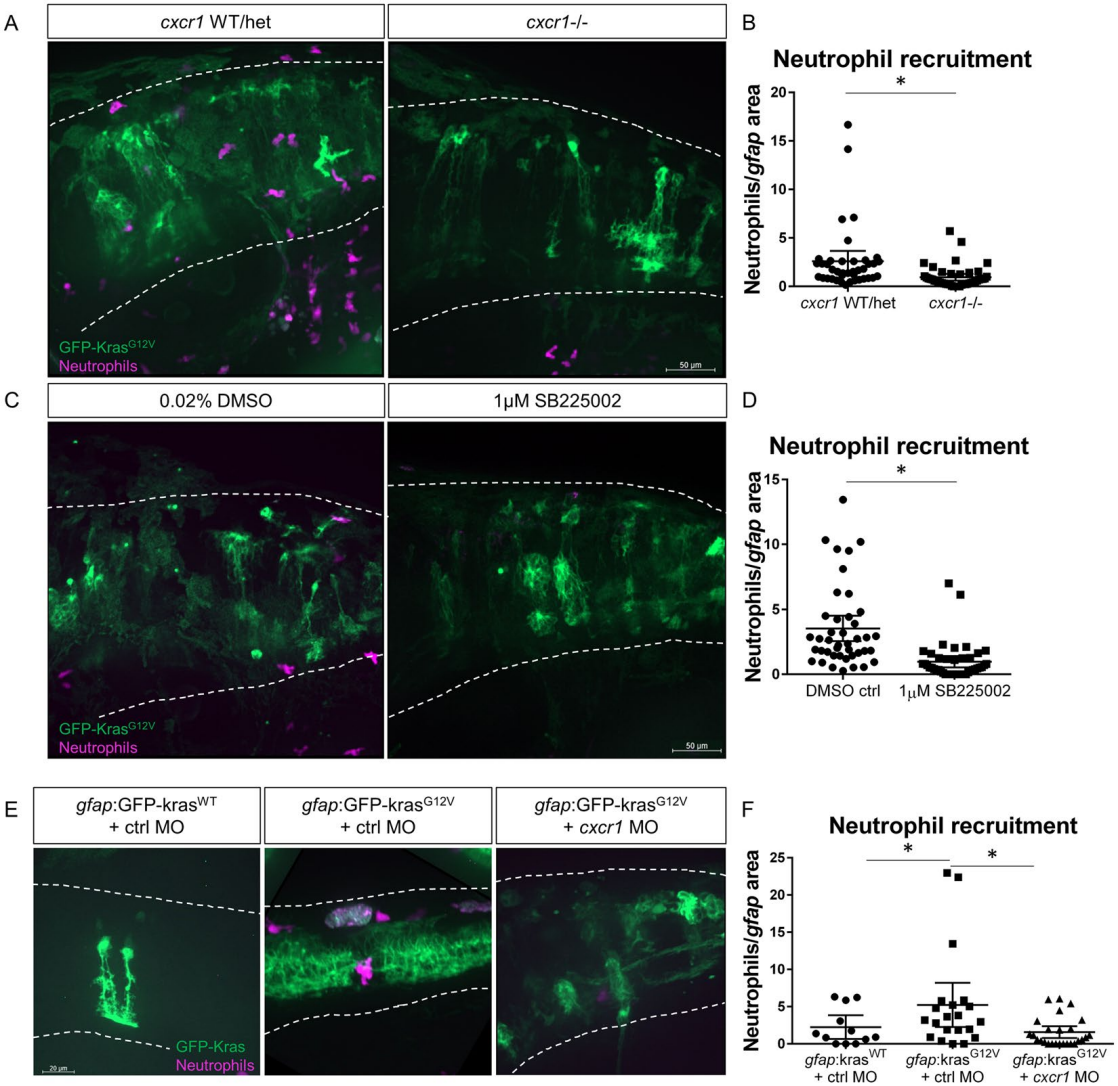
Cxcr1 promotes neutrophil recruitment to the tumor-initiating microenvironment. Adult *cxcr1* heterozygote zebrafish expressing Tg(*mpx*:mCherry) were in-crossed, injected with *gfap*:kras^G12V^, imaged for neutrophil recruitment to the hindbrain at 3 dpf, and then processed for single-embryo genotyping (**A**). Neutrophils recruited to the field of view were quantified in *cxcr1* mutants and WT or heterozygote siblings (no difference observed between +/+ and +/−) and normalized to total *gfap*-expressing area (**B**, n = 40 WT/het and 44 *cxcr1* mutant larvae). Tg(*mpx*:mCherry) larvae expressing *gfap*:kras^WT^ or kras^G12V^ were treated beginning at 2.5 dpf with 1 µM SB225002 or 0.02% DMSO vehicle control for 16 hours followed by live-imaging (**C**). Neutrophil recruitment was quantified and normalized as above (**D**, n = 42 DMSO and 43 SB225002 larvae). Wildtype Tg(*mpx*:mCherry) embryos were co-injected with *gfap*:GFP-kras^WT^ or kras^G12V^ and standard control MO or *cxcr1* splice-blocking morpholino. Larvae were live-imaged at 3 dpf (**E**) and neutrophil recruitment was analyzed as described previously (F, n = 12 kras^WT^, 21 kras^G12V^ ctrl MO, and 26 kras^G12V^ *cxcr1* MO larvae). *p < 0.05, One-way ANOVA with post-hoc Tukey’s test performed for data in 4 F.

As our data indicate that neutrophils support proliferation of transformed astrocytes, we next sought to investigate the signals responsible for neutrophil recruitment to the tumor-initiating niche. Our previous data showed that IL-8 family chemokines were upregulated with expression of oncogenic Kras in the brain (Fig. 2D), and similarly, IL-8 is upregulated in human glioblastoma^36^. We have also previously shown that zebrafish Cxcr1 is required for neutrophil recruitment to sterile wounds but not to infection and is dispensable for normal neutrophil development and random motility^24^. Therefore, we characterized the role of the IL-8 receptor Cxcr1 in regulating neutrophil recruitment to tumor-initiating cells. We injected *gfap*:GFP-Kras^G12V^ in wildtype, *cxcr1* +/−, or *cxcr1*−/− embryos expressing *mpx*:mCherry and then analyzed neutrophil recruitment to the hindbrain at 3 dpf (Fig. 4A). Similar to what was observed in the wounding microenvironment, we found that significantly fewer neutrophils were recruited to the hindbrain and surrounding tissue in *cxcr1* mutants compared to wildtype and *cxcr1* heterozygote siblings (Fig. 4B). Intriguingly, in mutants for the related receptor Cxcr2, we did not observe any decrease in neutrophil recruitment to transformed astrocytes (Figure S1), further suggesting the tumor-initiating microenvironment is similar to the wound neutrophil chemotactic microenvironment^24^. To determine if this recruitment mechanism could also be blocked by pharmacological inhibition, we used SB225002, an inhibitor of Cxcr1 and Cxcr2. In comparison to vehicle control, SB225002 treatment resulted in significantly fewer neutrophils recruited to the hindbrain of larvae expressing *gfap*:Kras^G12V^ (Fig. 4C,D) thus recapitulating the Cxcr1 mutant phenotype. Additionally, transient knockdown of *cxcr1* with splice-blocking morpholino similarly reduced neutrophil recruitment to the *gfap*:Kras^G12V^-expressing hindbrain (Fig. 4E,F). In a related model of epithelial cell transformation in which Kras^G12V^ was transiently expressed in larval zebrafish keratinocytes, neutrophil recruitment was also reduced upon knockdown of *cxcr1* (data not shown). These data demonstrate the requirement of Cxcr1 signaling, but not Cxcr2, in neutrophil trafficking to the tumor-initiating microenvironment, similar to the wound microenvironment.

### Cxcr1/2 inhibition reduces proliferation and progression of tumor-initiating astrocytes

**Figure 5:**
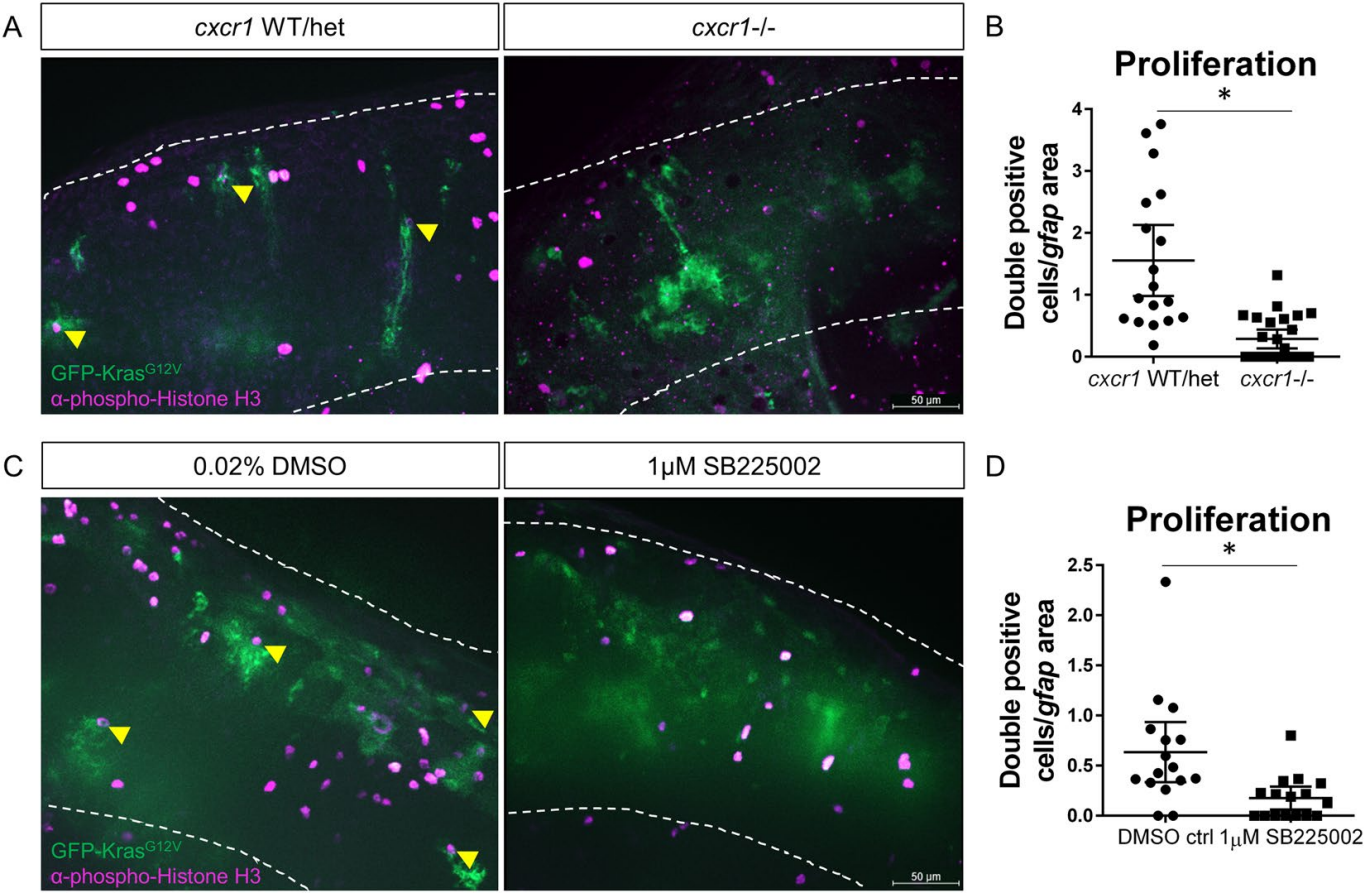
Blocking neutrophil chemotaxis signaling reduces proliferation of tumor-initiating cells. *cxcr1* heterozygote fish were in-crossed, injected with *gfap*:kras^G12V^, fixed for pH3 immunostaining at 3 dpf, and genotyped post-imaging as described above (**A**). pH3-GFP double-positive cells were quantified and normalized as in previous figures (**B**, yellow arrowheads in **A**, n = 19 WT/het and 26 *cxcr1*−/− larvae). Wildtype larvae expressing *gfap*:kras^G12V^ were treated with 1 µM SB225002 or DMSO control as described in Fig. 4. Larvae were fixed at 3 dpf and immunostained for phospho-Histone H3 (**C**). Proliferating cells were quantified as above (**D**, double-positive cells marked by yellow arrowhead in C, n = 17 larvae/condition). *p < 0.05.

We next investigated the effect of Cxcr1/2 inhibition on the proliferation of tumor-initiating astrocytes. Similar to what we observed in larvae with impaired neutrophil motility, we observed a significant reduction in proliferating Kras-positive astrocytes in *cxcr1* mutant larvae compared to wildtype or heterozygous siblings (Fig. 5A,B). Importantly, we observed the same phenotype upon pharmacological inhibition of Cxcr1/2 signaling with SB225002 treatment. In *gfap*:Kras^G12V^-expressing larvae treated with SB225002, proliferation of transformed astrocytes was significantly reduced compared to vehicle control (Fig. 5C,D). To determine if the reduction in proliferation observed in the SB225002-treated larvae was attributable primarily to neutrophil loss in the tumor-initiating microenvironment, we next treated neutrophil-specific Rac2^D57N^ mutants with SB225002 or DMSO control. At 3 dpf, no additive effect was observed in Rac2^D57N^ mutants treated with SB225002 compared to DMSO controls (Figure S2), suggesting that the proliferation reduction observed in SB225002 is primarily due to the lack of neutrophils in the microenvironment and not due to other cell types within the microenvironment or astrocyte-intrinsic effects. These data demonstrate that Cxcr1/2 promotes proliferation of tumor-initiating astrocytes and suggests that this proliferation is due to Cxcr1/2-mediated neutrophil recruitment.

### Oncogenic Kras induces neoplastic masses in *p53*−/− larvae which requires Cxcr1/2 signaling

**Figure 6:**
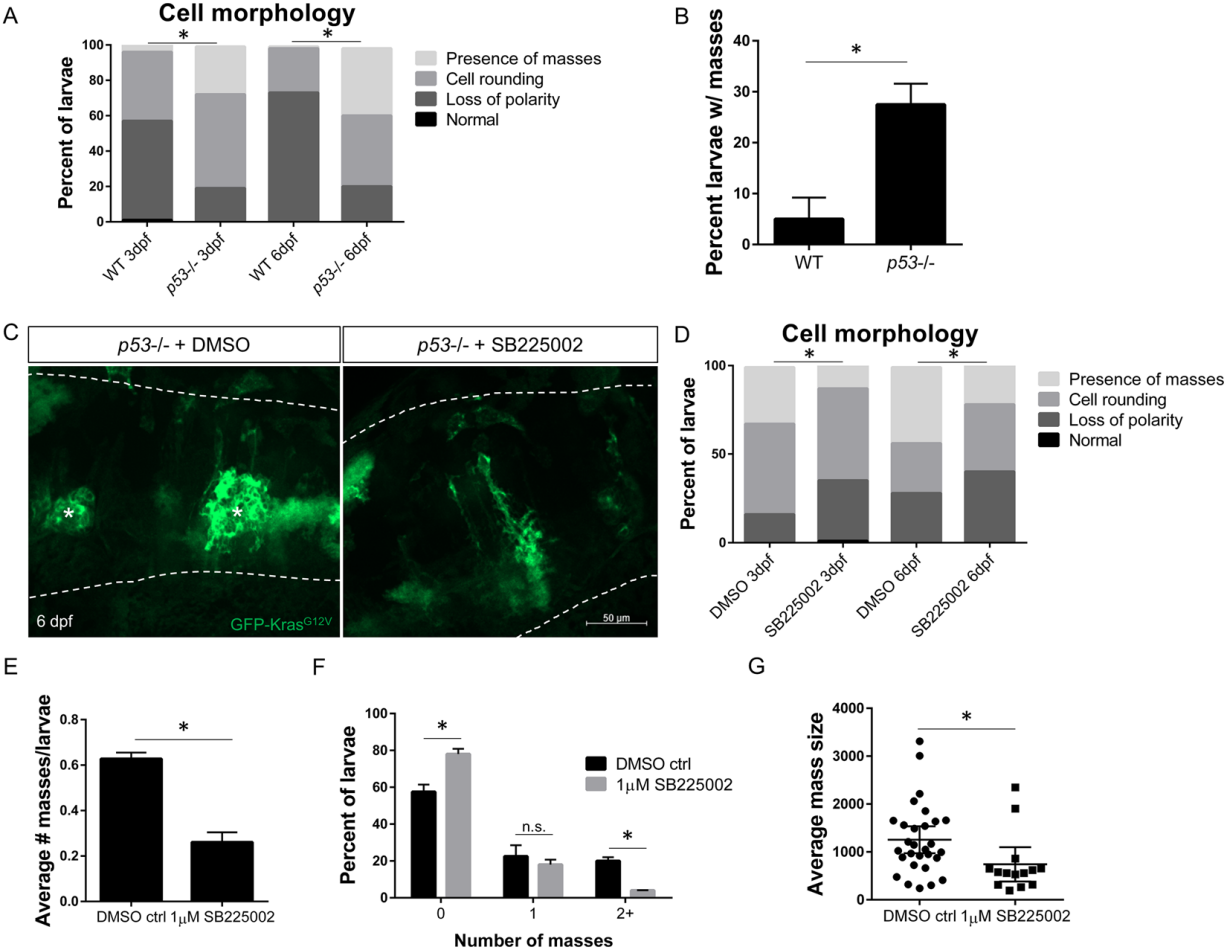
Inhibition of neutrophil chemotaxis reduces early mass formation in *p53* mutant larvae. Morphology of *gfap*:GFP-kras^G12V^ expressing astrocytes in WT or *p53* mutant background at 3 and 6 dpf (**A**, n = 87 WT 3dpf, 113 *p53*−/− 3dpf, 75 WT 6 dpf, and 99 *p53*−/− 6dpf larvae; Chi-squared analysis performed). The percentage of larvae developing masses at 6 dpf is shown in (**B**). *p53* mutant embryos were injected with *gfap*:GFP-kras^G12V^ plasmid and treated with DMSO control or 1uM SB225002 beginning at 2.5 dpf with drug refreshed every 24 hrs. Larval hindbrain was live-imaged at 3 dpf and 6 dpf (6 dpf shown in (**C**), asterisks denote masses). Morphology of GFP-expressing cells was categorized as described in Fig. 1B (**D**, n = 84 DMSO 3 dpf, 88 SB225002 3 dpf, 39 DMSO 6 dpf, and 50 SB225002 6 dpf larvae; Chi-squared analysis performed). Average number of masses was quantified in all larvae at 6 dpf (**E**, n = 40 DMSO and 50 SB225005 larvae). Larvae were also categorized by number of masses formed (0,1, or ≥ 2) by 6 dpf (**F**) and in larvae with masses present, average mass size (μm^2^) was measured (**G**, n = 29 DMSO and 15 SB225002 larvae). *p < 0.05 For Supplementary Figure Legends, please see Supplementary Information file.

To determine the effect of Cxcr1/2 signaling on the outcome of transformed cells, we turned to the more tumor-permissive *p53* mutant background which allows for heightened progression of disease in other zebrafish cancer models^37–40^. We found that *p53*−/− larvae expressing *gfap*:Kras^G12V^ exhibit more advanced tumor-initiation at 3 and 6 dpf with a higher proportion of larvae demonstrating cell rounding and mass formation compared to the wildtype background (Fig. 6A,B). Importantly, recruitment of neutrophils to these masses was normal compared to wildtype background, as was neutrophil migration to a tail-fin wound, suggesting that *p53* mutants have normal neutrophil motility and behavior (Figure S3). This enhanced and more rapid mass growth in the *p53* mutants allowed us to assay whether blocking neutrophil chemotaxis to the tumor-initiating niche affects early tumorigenesis beyond proliferation. *p53* mutant larvae expressing *gfap*:Kras^G12V^ were treated with the Cxcr1/2 inhibitor SB225002 starting at 2.5 dpf, with drug or vehicle refreshment every 24 hours, and imaged on 3 dpf and 6 dpf for astrocyte morphology (Fig. 6C,D). As expected based on the reduced proliferation of transformed cells observed in SB225002-treated WT larvae, mass formation was also significantly reduced when Cxcr1/2 was blocked in *p53* mutants (Fig. 6D,E). Concurrently, a higher proportion of transformed astrocytes exhibited the less severe morphology of altered polarity, demonstrating that while blocking Cxcr1/2 signaling is not able to fully restore Kras^G12V^-expressing cells to their normal, wildtype morphology, it does appear to inhibit progression to more advanced stages of cellular transformation. Similarly, mass number was significantly inhibited in SB225002-treated *p53*−/− larvae with almost no larvae developing 2 or more masses compared to approximately 20% of control-treated larvae (Fig. 6F). Additionally, in the larvae that developed masses, the average mass size was significantly decreased in SB225002-treated larvae compared to controls (Fig. 6G). Altogether, these data suggest that blocking Cxcr1/2 inhibits tumor initiation and progression of neoplastic astrocytes *in vivo*, further supporting a role for Cxcr1 signaling at the tumor-forming niche.

## Discussion

Here we demonstrate that neutrophils play a tumor-promoting role in the early tumor microenvironment within the brain using transparent zebrafish larvae. In this model of glioblastoma initiation, we show that neutrophils are actively recruited to tumor-initiating cells very early in oncogenesis. The presence of neutrophils within the microenvironment enhances proliferation of oncogene-transformed cells. We demonstrate a role for the chemokine receptor Cxcr1 in the neutrophil response to transformed astrocytes. Interestingly, Cxcr2 does not appear to be required for neutrophil recruitment to the tumor-initiating microenvironment, suggesting that neutrophil response to the early TME is more like their response to wounds than infected tissue^24,31^. We also find that pharmacological inhibition of the Cxcr1/2 signaling pathway decreases neutrophil recruitment, reduces early proliferation of tumor-initiating cells, and reduces subsequent pre-tumorous mass formation in a *p53* null background.

A role for the IL8-CXCR1/2 signaling axis has been previously demonstrated in cancer and other inflammatory diseases^11^. Specifically in glioma, high expression of CXCR2 correlates with high-grade glioma in patients, poor prognosis, and tumor recurrence, while inhibition of CXCR1/2 signaling with the SB225002 inhibitor reduced migration of glioma cells *in vitro*^41^. Here, we show that in addition to tumor cell-intrinsic roles for Cxcr1/2, this signaling pathway is also crucial for recruitment of neutrophils into the tumor-initiating microenvironment which then supports proliferation of transformed cells. Indeed, we observed no additive effect of Cxcr1/2 inhibition on proliferation in neutrophil-deficient lines, suggesting that Cxcr1/2 inhibition primarily affects transformed astrocyte proliferation via effects on neutrophils rather than through astrocyte-intrinsic pathways in this model. Importantly, pharmacological inhibitors of CXCR1/2 are already in clinical trials for several inflammatory diseases including COPD, asthma, and rheumatoid arthritis as well as for certain cancers including breast cancer and melanoma^11,42^.

Previous data from our lab suggests that Cxcr1 is specifically required for neutrophil recruitment to sites of sterile tissue damage and is dispensable for recruitment to infection^24^. This type of specificity for the TME and not for infected tissue is particularly useful for cancer patients since chemotherapy-related neutropenia increases infection risk in these patients^43^. Our data suggest that specific inhibition of CXCR1 may be a potential target of interest in glioblastoma and other tumor types that are characterized by high levels of neutrophil infiltration or alternatively as a potential prophylactic measure for patients at high risk of developing inflammation-associated cancers. It is interesting that the other zebrafish Cxcl8 receptor Cxcr2 was not required for recruitment to transformed astrocytes, although it is necessary for neutrophil recruitment to infection^31^. These findings support the TME as a type of “unhealed wound” with the involvement of similar environmental cues and receptors that mediate neutrophil infiltration. Recently, mouse Cxcr2 was demonstrated to play a role in neutrophil accumulation within the tumor as well as T cell infiltration and tumor metastasis^44^. Interestingly, the zebrafish model suggests that neutrophils can play a role in tumor progression independent of T cells. It is also important to note that it is unclear whether zebrafish Cxcr1 and Cxcr2 are functionally homologous to their mammalian orthologs. Evidence suggests that in zebrafish, Cxcr1 and Cxcr2 play distinct roles in regulating neutrophil recruitment to different tissue insults. Whether these distinct roles are conserved in humans remains unclear.

Tumor associated neutrophils are an intriguing target for the treatment of human cancer. In humans, neutrophils have been implicated in cancer progression in several cancers. Our data suggest that neutrophils can support proliferation of early tumor-initiating astrocytes. These findings are consistent with the idea that chronic neutrophil inflammation can lead to increased risk of developing cancer^45,46^. One explanation for how neutrophil exert their effect is that neutrophils produce reactive oxygen and nitrogen species (ROS and RNS) which can cause DNA damage and may induce oncogenesis in nearby cells. Our data suggest that, in addition, the presence of neutrophils promotes proliferation of transformed cells in the brain. Our findings are consistent with previous studies which have demonstrated that in zebrafish models of skin oncogenesis, neutrophil recruitment enhances proliferation and EMT of transformed cells^28,47,48^. One of the potential mechanisms for this interaction is through the production of neutrophil elastase (NE), which has been shown to enhance proliferation of cancer and other cell types in culture^49–53^. Indeed, inhibitors of NE have previously been suggested as potential anti-cancer therapeutic targets by several groups^54–57^. In addition to their demonstrated role in the primary TME, neutrophils have also been implicated in priming the metastatic niche for secondary tumor growth^58^. Our data suggest that targeting neutrophil chemotaxis and tumor infiltration could provide a useful avenue for developing future clinical targets. One exciting new approach is exploiting neutrophils’ highly migratory and tumor-infiltrative capabilities to target drugs to the TME^59^. In a recent study by Xue *et al*., primary mouse neutrophils loaded with liposomes containing paclitaxel were injected into the blood stream of a glioma mouse model post-tumor resection, resulting in neutrophil homing to the brain, release of the drug, and ultimately increased survival in treated mice. Further understanding of the signals and receptors which enhance neutrophil recruitment into the TME could allow the engineering of drug-delivering neutrophils with even greater TME homing and tumoricidal efficacy.

There is some controversy over whether TANs are generally tumor-promoting or whether they can also play a role in killing and clearance of tumor cells. There is evidence that, similar to macrophages, neutrophils can be polarized into an anti-tumor N1 or pro-tumor N2 phenotype depending on signals from the microenvironment such as TGF-β^8^. There are also several subpopulations of neutrophils that display a variety of characteristics and phenotypes which likely can contribute to a heterogeneity of TAN behavior and impact^10^. Exposure to the TME itself may also influence neutrophil plasticity and polarization, adding further complexity to understanding the role of neutrophils in cancer progression^9,60^. Importantly, little is known about how chemotactic signaling receptors are regulated throughout neutrophil recruitment and plasticity in response to tumor formation and if different neutrophil subpopulations employ different pathways to regulate and direct their migration. Further study is needed to determine the migratory mechanisms and behavior of tumor-activated neutrophils, as well as their role in cancer progression in a variety of cancer types to inform future therapies to treat human cancer.

## Electronic supplementary material

Supplementary Information

Supplemental Movie 1

Supplemental Movie 2

## Data Availability

All data generated or analysed during this study are included in this published article (and its Supplementary Information files). No datasets were generated or analysed during the current study.
