# Supplementary material for: Cxcr1 mediates recruitment of neutrophils and supports proliferation of tumor-initiating astrocytes in vivo
Source: Sci Rep. 2018 Sep 5;8:13285. doi: 10.1038/s41598-018-31675-0 (PMC6125480; doi:10.1038/s41598-018-31675-0)
Supplement: Supplementary file 1 — Supplementary Information [file 41598_2018_31675_MOESM1_ESM.pdf]

Title: Cxcr1 mediates recruitment of neutrophils and supports proliferation of tumor-initiating astrocytes *in vivo*

Authors: Davalyn Powell[1], Meng Lou[1], Francisco Barros Becker[1], and Anna Huttenlocher[1,2*]

[1]Department of Medical Microbiology and Immunology, [2]Department of Pediatrics, University of Wisconsin-Madison, Madison, WI, USA.

*Corresponding author: huttenlocher@wisc.edu

# Supplementary Information

Includes:

Supplemental Figures 1-3

Supplemental Figure Legends

Supplemental Movie Legends

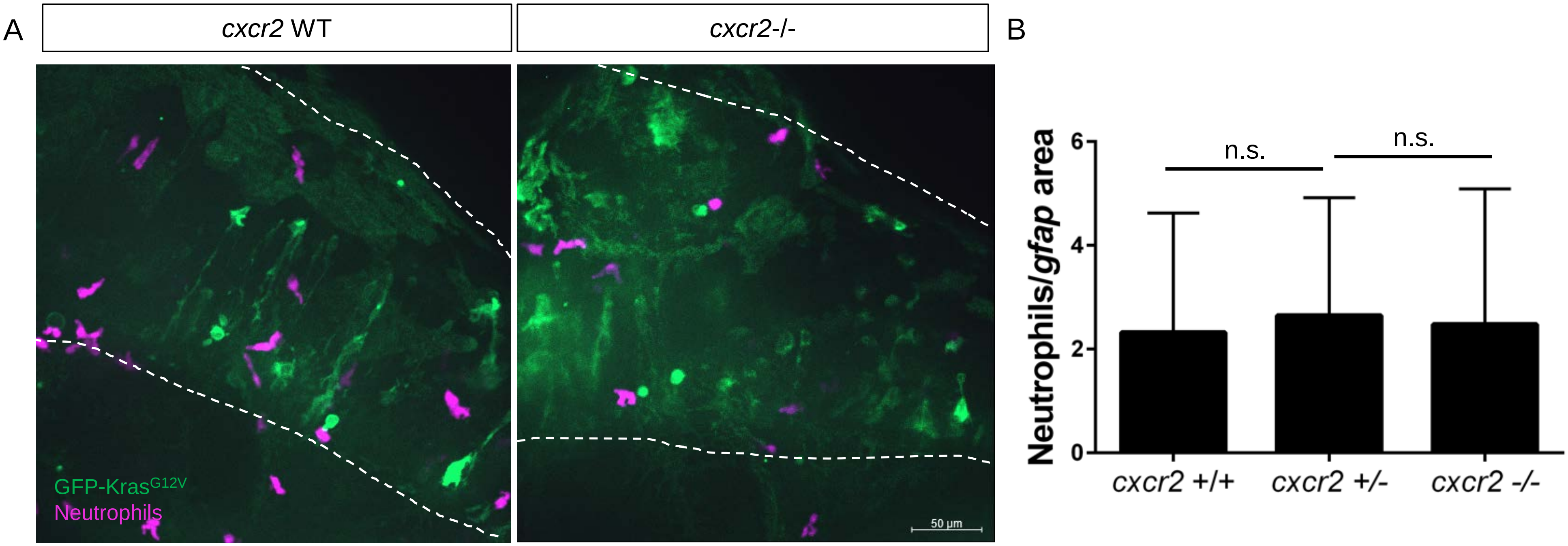


Supplemental Figure 1

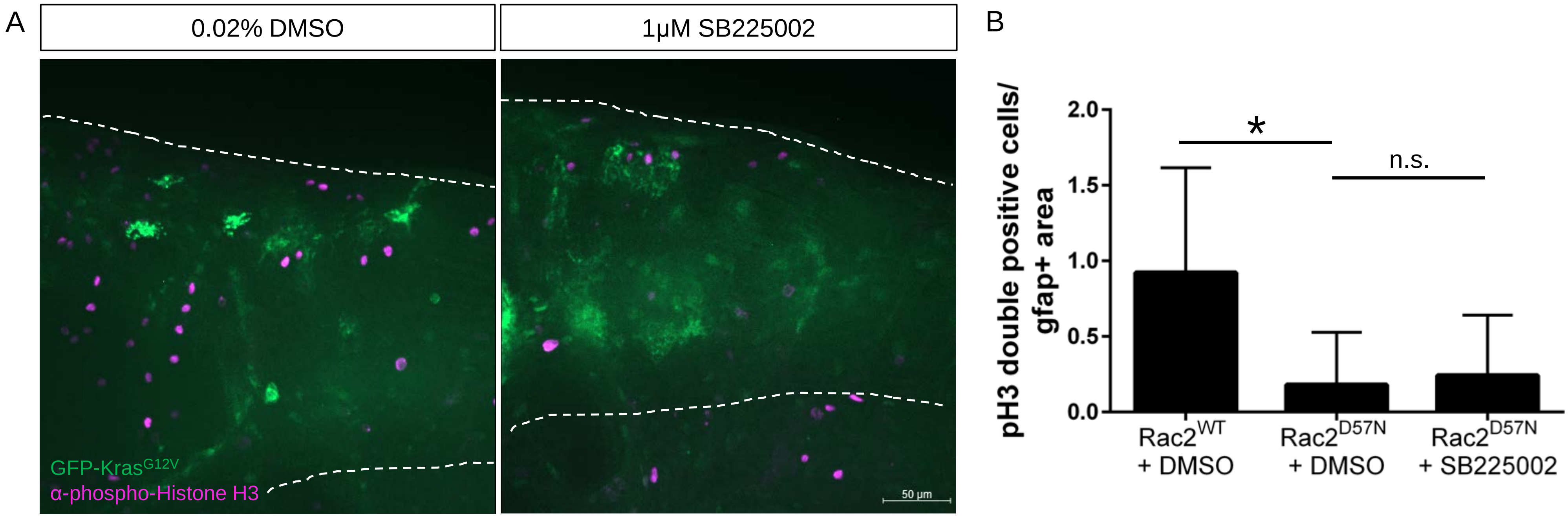


Supplemental Figure 2

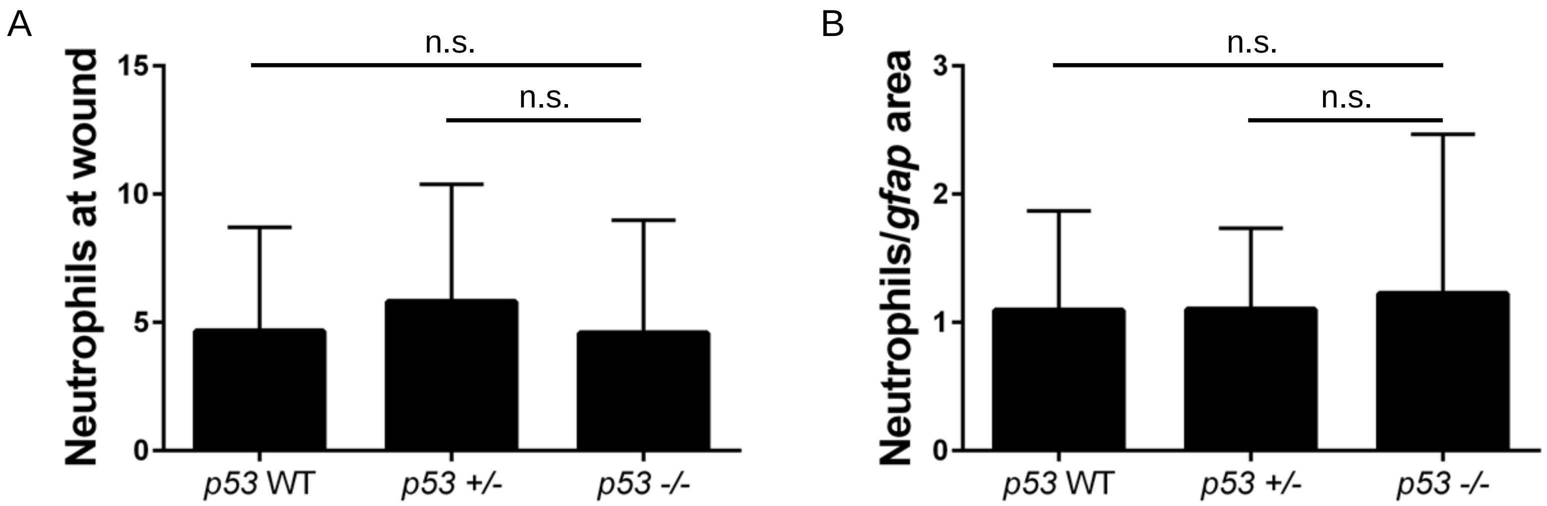


Supplemental Figure 3

Supplemental Figure Legends:

**Supplemental Figure 1. Cxcr2 is dispensable for neutrophil recruitment to tumor-initiating astrocytes.** Adult *cxcr2* heterozygote zebrafish expressing Tg(*mpx*:mCherry) were in-crossed, injected with *gfap*:kras$^{G12V}$, imaged for neutrophil recruitment to the hindbrain at 3 dpf, and then processed for single-embryo genotyping (A). Neutrophils recruited to the field of view were quantified in *cxcr2* mutants and WT or heterozygote siblings (no difference observed between +/+ and +/-) and normalized to total *gfap*-expressing area (B, n= 24 WT, 23 *cxcr2*+/-, 7 *cxcr2*-/-). Data pooled from 2 replicates.

**Supplemental Figure 2. Cxcr1/2 inhibition with neutrophil-specific motility loss.**

Tg(*mpx*:mCherry-2a-Rac2$^{WT}$) or Tg(*mpx*:mCherry-2a-Rac2$^{D57N}$) embryos were injected with *gfap*:GFP-kras$^{G12V}$ and treated with 1µM SB225002 or DMSO control as described in Figure 4. Larvae were fixed at 3 dpf and immunostained for phospho-Histone H3 (A). pH3-GFP double positive cells were quantified by optical section and normalized as above (B, n= 10 Rac2$^{WT}$ + DMSO, 19 Rac2$^{D57N}$ + DMSO, 17 Rac2$^{D57N}$ + SB225002). Data pooled from 2 replicates, *$p<0.05$.

**Supplemental Figure 3. Neutrophil recruitment is normal in *p53* mutant background.** Adult *p53* heterozygote zebrafish expressing Tg(*mpx*:mCherry) were in-crossed, exposed to a tail-transection wound at 3 dpf, then imaged 2 hours later for neutrophil recruitment to the wound edge followed by single embryo genotyping. Neutrophils caudal to the notochord were quantified (A). *p53* heterozygote zebrafish expressing Tg(*mpx*:mCherry) were in-crossed, injected with *gfap*:kras$^{G12V}$, imaged for neutrophil recruitment to the hindbrain at 3 dpf, and then processed for single-embryo

genotyping. Neutrophils recruited to the field of view were quantified and normalized to total *gfap*-expressing area (B). Data pooled from 2 replicates.

Supplemental Movie Legends:

**Supplemental Movie 1. Time-lapse imaging of neutrophil recruitment to *gfap*:Kras$^{WT}$ cells.** Live-imaging of neutrophils in the hindbrain of Tg(*mpx*:mCherry) larvae expressing *gfap*:GFP-kras$^{WT}$. Time-lapse imaging by spinning disc confocal microscope was performed for 9 hours beginning at 3 dpf. Total neutrophils (magenta) recruited to the hindbrain during the period of imaging were quantified in Figure 2C.

**Supplemental Movie 2. Time-lapse imaging of neutrophil recruitment to *gfap*:Kras$^{G12V}$ cells.** Live-imaging of neutrophils in the hindbrain of Tg(*mpx*:mCherry) larvae expressing *gfap*:GFP-kras$^{G12V}$. Time-lapse imaging by spinning disc confocal microscope was performed for 9 hours beginning at 3 dpf. Total neutrophils (magenta) recruited to the hindbrain during the period of imaging were quantified in Figure 2C.
